# Robotic-Assisted Sigmoidectomy with D3 Lymphadenectomy for Sigmoid Colon Cancer in a Patient with Situs Inversus Totalis: A Case Report

**DOI:** 10.70352/scrj.cr.25-0588

**Published:** 2026-01-29

**Authors:** Kazuhito Minami, Shojiro Kawano, Tomohiro Furukawa, Yuriko Tsutsui, Koichi Kimura, Yoshinari Nobutou, Hiroko Yano, Yuichiro Kajiwara, Ryosuke Minagawa, Takashi Nishizaki

**Affiliations:** Department of Surgery, Matsuyama Red Cross Hospital, Matsuyama, Ehime, Japan

**Keywords:** situs inversus totalis, robotic surgery, sigmoid colon cancer, D3 lymphadenectomy

## Abstract

**INTRODUCTION:**

Situs inversus totalis (SIT) is a rare congenital anomaly characterized by complete mirror-image transposition of thoracic and abdominal organs. Although laparoscopic colectomy in SIT has been increasingly reported, the reversed anatomy and inherent limitations of laparoscopy make such procedures technically demanding. Robotic-assisted surgery may overcome these issues by providing enhanced ergonomics and intuitive instrument control, yet reports in SIT remain scarce.

**CASE PRESENTATION:**

A 51-year-old man with SIT presented with anal bleeding and was diagnosed with sigmoid colon cancer. Preoperative CT confirmed complete visceral inversion without distant metastasis. Robotic-assisted sigmoidectomy with D3 lymphadenectomy was performed using the da Vinci Xi surgical system. A patient-specific port configuration was designed based on preoperative imaging, minimized arm collisions, and optimized instrument mobility. The procedure was uneventful, with an operative time of 232 minutes and minimal blood loss. The patient was discharged on POD 7 without complications. Histopathological examination revealed pT3, pN1a, cM0, Stage IIIB adenocarcinoma.

**CONCLUSIONS:**

This case demonstrates that robotic-assisted colectomy can effectively overcome the ergonomic and technical challenges posed by SIT. Patient-specific port mapping, guided by preoperative imaging, is essential to maximize the benefits of robotic technology. Furthermore, this report contributes to the limited evidence supporting robotic colectomy as a feasible and advantageous approach for patients with SIT.

## Abbreviations

ASIS
: anterior superior iliac spine
CA19-9
: carbohydrate antigen 19-9
CEA
: carcinoembryonic antigen
ECOG
: Eastern Cooperative Oncology Group
R0
: complete resection with no residual tumor
SIT
: situs inversus totalis

## INTRODUCTION

SIT is a rare congenital anomaly with an incidence of approximately 1 in 5000–10000 individuals.^1)^ Although SIT itself does not increase malignancy risk, its mirror-image anatomy poses unique technical challenges in abdominal surgery. In recent years, laparoscopic resections for gastrointestinal malignancies in SIT have been increasingly reported.^2–6)^ These reports highlight the need for modified port placement, altered team positioning, and meticulous preoperative planning. Nevertheless, the intrinsic limitations of laparoscopy—including suboptimal ergonomics, restricted instrument articulation in confined spaces, and increased risk of instrument collisions—remain difficult to overcome.^2–4)^

Robotic-assisted surgery offers potential solutions. With articulated EndoWrist instruments (Intuitive Surgical, Sunnyvale, CA, USA), stable surgeon-controlled retraction, and 3D high-definition visualization, robotic platforms allow precise dissection and intuitive motion translation without the need for mental reversal of anatomy.^7–9)^ Such advantages may be particularly beneficial in SIT, where conventional laparoscopy demands constant cognitive adjustment. However, published reports of robotic colectomy in SIT remain scarce.

Herein, we describe a rare case of robotic-assisted sigmoidectomy with D3 lymphadenectomy for sigmoid colon cancer in a patient with SIT, emphasizing technical adaptations, procedural feasibility, and clinical implications.

## CASE PRESENTATION

**Fig. 1:**
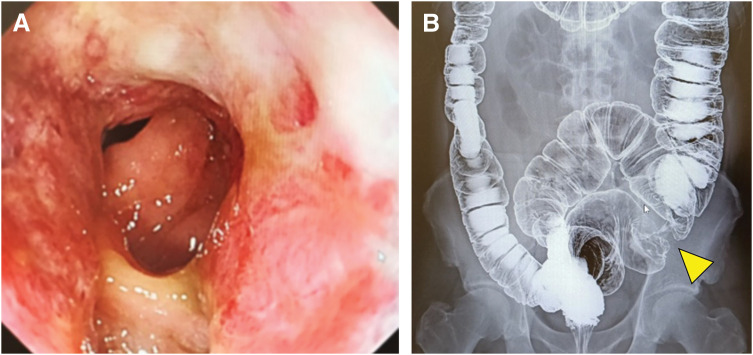
(**A**) Colonoscopic image showing an elevated lesion in the sigmoid colon. (**B**) Barium enema showing an irregular stenotic lesion, the arrowhead indicates the primary tumor.

**Fig. 2:**
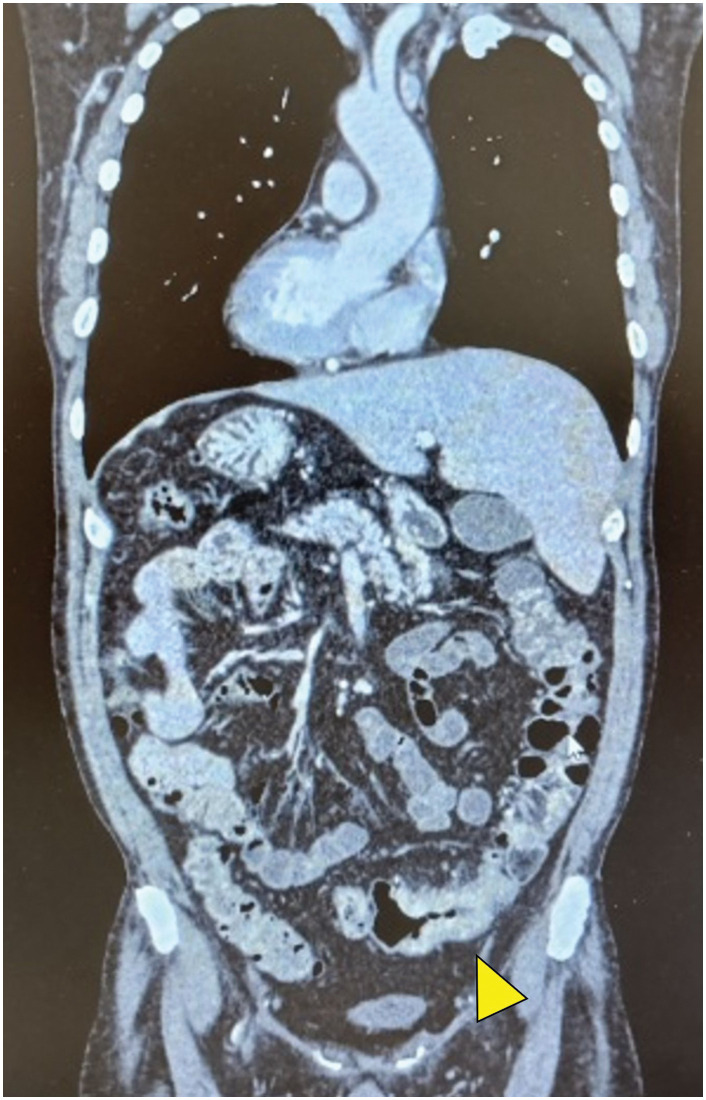
Contrast-enhanced CT showing complete mirror-image anatomy consistent with situs inversus totalis. The liver is on the left and the spleen on the right. CT image demonstrating wall thickening of the sigmoid colon (arrowhead), corresponding to the primary tumor.

A 51-year-old man presented with anal bleeding. Colonoscopy revealed a 5-cm advanced sigmoid colon tumor (**Fig. 1A**, **1B**), and biopsy confirmed well-differentiated tubular adenocarcinoma. Barium enema delineated the lesion, and thoracoabdominal CT demonstrated complete visceral inversion without lymphadenopathy or distant metastasis (**Fig. 2**). Laboratory findings, including CEA and CA19-9, were within normal limits (**Table 1**). His BMI was 27 kg/m^2^, ECOG performance status was 0, and past medical history was unremarkable.

**Table 1:** Laboratory findings including tumor markers

White blood cell (×10^2^/μl)	118.9	Na (mEq/L)	141
Hb (g/dl)	15.4	K (mEq/L)	4.5
Plate (×10^4^/μl)	32.6	Cl (mEq/L)	105
Total Protein (g/dl)	7.3	Total Cholesterol (mg/dl)	247
Albumin (g/dl)	4.6	Triglycerides (mg/dl)	92
Total Bilirubin (mg/dl)	1.1	FBS (mg/dl)	102
AST (U/L)	23	CRP (mg/dl)	0.1>
ALT (U/L)	31	PT-INR	0.97
LDH (U/L)	185	APTT (sec)	31.6
BUN (mg/dl)	14	CEA (ng/ml)	4.8
Creatinine (mg/dl)	1.06	CA19-9 (U/ml)	3.7

Robotic-assisted sigmoidectomy with D3 lymphadenectomy was performed using the da Vinci Xi surgical system (Intuitive Surgical Sunnyvale, CA, USA). Preoperative contrast-enhanced CT with 3D reconstruction of the mesenteric vessels and colon was reviewed to understand the mirror-image vascular anatomy and to simulate the working range and angles of each robotic arm, particularly around the inferior mesenteric artery (IMA) origin and within the deep pelvis. The patient was placed in lithotomy position with steep Trendelenburg and left-side-down tilt.

**Fig. 3:**
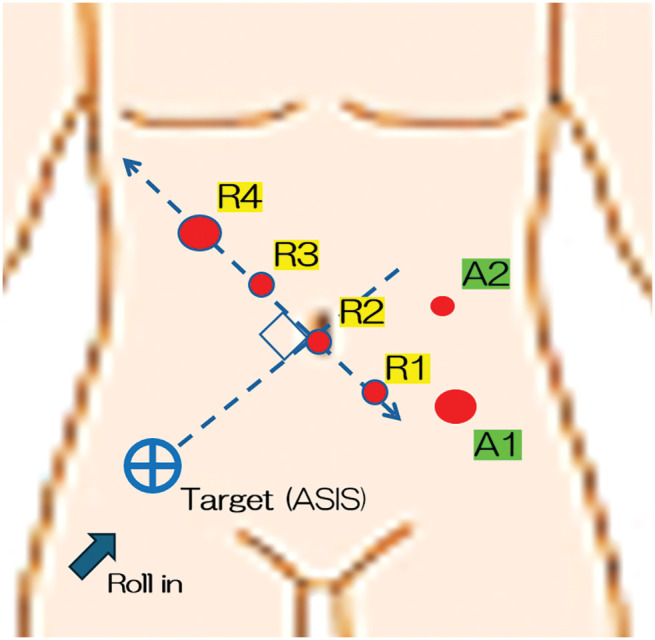
Target marker at the right ASIS. A line was drawn between the umbilical camera port (R2) and the ASIS, and a perpendicular reference line was set from the right upper abdomen to the left lower abdomen. Ports R1–R4 were placed along this line to minimize arm interference. ASIS, anterior superior iliac spine

Port placement: Based on these 3D CT findings, with the target marker set at the right ASIS, we drew a line connecting the umbilical camera port (R2) to the ASIS and established a perpendicular reference line from the right upper abdomen to the left lower abdomen. Robotic ports were placed along this perpendicular line at 6–8 cm intervals to minimize robotic arm collisions: an 8-mm umbilical camera port (Arm 2, 30° scope); an 8-mm right upper quadrant port for monopolar scissors (Arm 3); an 8-mm left lower quadrant port for fenestrated bipolar forceps (Arm 1); a 12-mm port lateral to Arm 3 for a Tip-up fenestrated grasper or SureForm stapler (Arm 4); and assistant ports (A1: a 12-mm AirSeal in the left lower quadrant, positioned along the working axis to facilitate suction, stapler reload, clip application, gauze insertion, and specimen handling in the lower abdomen and pelvis; A2: a 5-mm port in the left upper quadrant dedicated to auxiliary retraction and irrigation while remaining outside the main arc of the robotic arms). The patient cart was rolled in from the right caudal side and docked with the boom oriented approximately parallel to the perpendicular reference line to further reduce arm interference (**Fig. 3**).

**Fig. 4:**
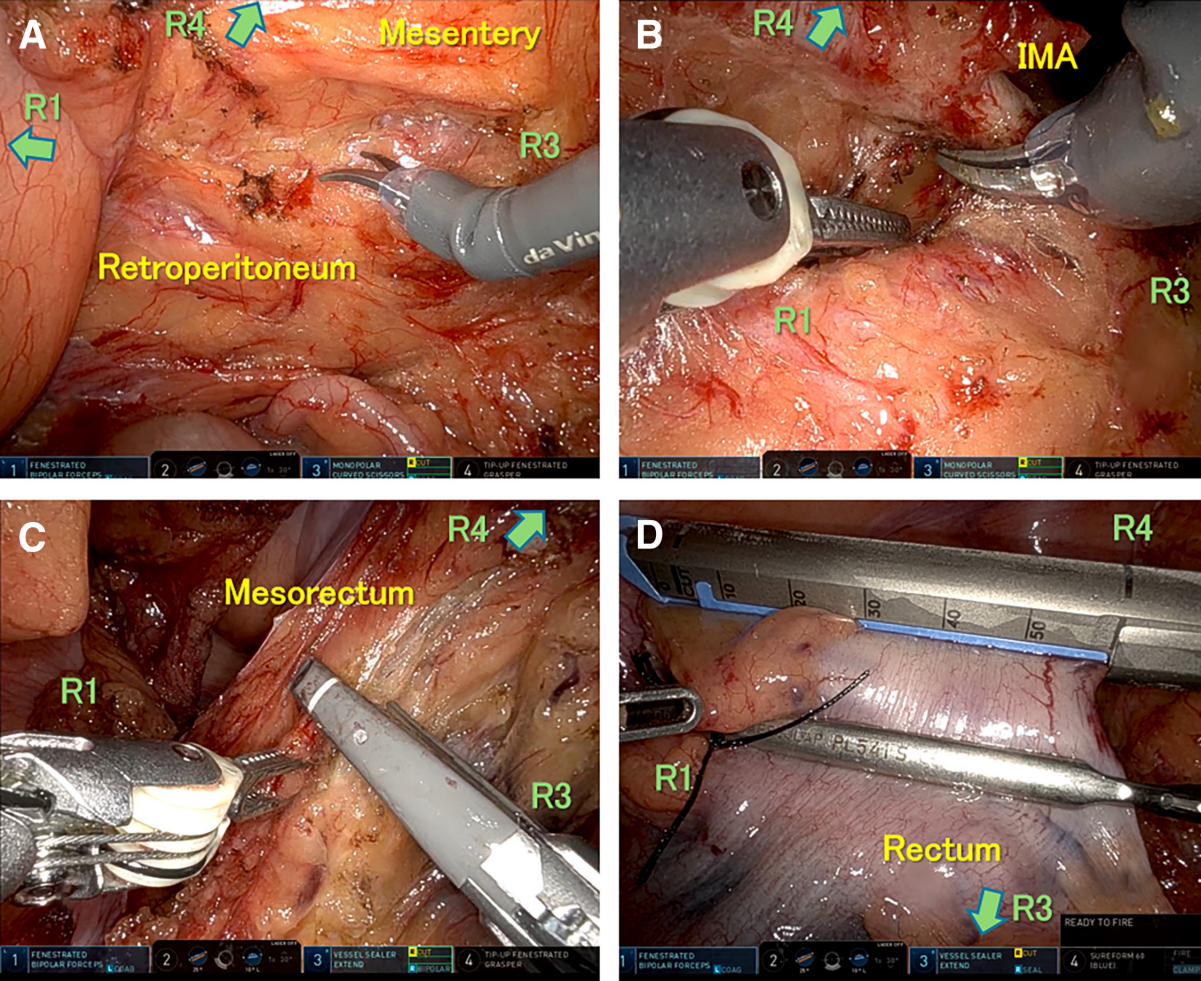
Robotic steps in SIT sigmoidectomy with regional lymphadenectomy. (**A**) Medial-to-lateral mesenteric mobilization with right ureter preservation. (**B**) IMA skeletonization and division at the origin. (**C**) Upper mesorectal dissection (da Vinci Vessel Sealer Extend, Arm 3) to secure distal margin. (**D**) Rectal transection (SureForm, Arm 4) and protected infraumbilical extraction. Arrows indicate traction vectors of R1–R4. IMA, inferior mesenteric artery; SIT, situs inversus totalis

Key robotic steps (corresponding to **Fig. 4A**–**4D**):

(a) Mesenteric mobilization: Medial-to-lateral dissection initiated at the sacral promontory with early identification and preservation of the right ureter in SIT. Dissection was performed with monopolar scissors (Arm 3) under counter-traction by the Tip-up grasper (Arm 4) while fenestrated bipolar forceps (Arm 1) maintained tissue control.
(b) Vascular control: Skeletonization and division of the inferior mesenteric artery at its origin (± division of the inferior mesenteric vein [IMV] as indicated for D3 lymphadenectomy), using Arm 3 with counter-traction by Arm 4 and hemostatic assistance by Arm 1.
(c) Distal mobilization: Upper mesorectal dissection to secure an adequate distal margin with autonomic nerve preservation, with Arm 4 providing upward traction and Arms 1/3 (using the da Vinci Vessel Selar Extend) dissecting along the mesorectal plane.
(d) Transection and retrieval: Rectal transection was performed using the SureForm stapler (Arm 4) with targeted traction from Arm 3. Although the stapler appears to approach the rectum at an oblique angle in **Fig. 4D**, the wristed articulation of Arm 4 allowed us to adjust the cartridge to be effectively perpendicular to the rectal axis at the time of firing. While the A1 assistant port could also have been used as an access route for stapling, we preferred the robot-controlled stapler via Arm 4 because it provided more stable, tremor-free motion than manual firing from the assistant side. The specimen was extracted through a protected infraumbilical incision.

**Fig. 5:**
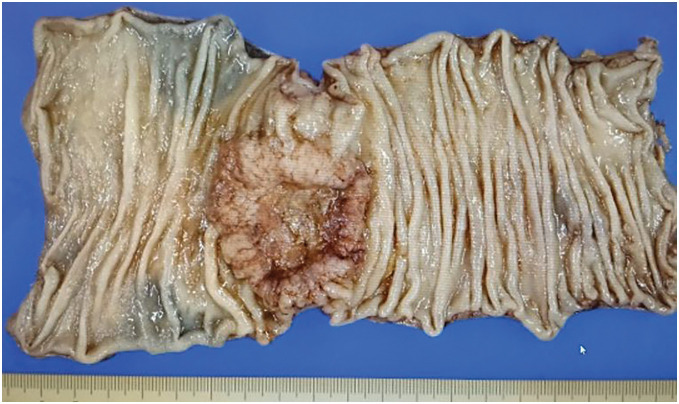
Resected specimen showing a 5-cm sigmoid colon carcinoma.

Surgeon-controlled retraction using the 4th arm maintained stable exposure throughout the D3 lymphadenectomy and pelvic dissection. Operative time was 232 min (console 154 min), with <5 mL blood loss. R0 resection was achieved. Gross examination confirmed a 5-cm tumor (**Fig. 5**). Fifteen lymph nodes were retrieved, consistent with an adequate D3 lymphadenectomy, and the proximal, distal, and radial margins were free of tumor (pPM0, pDM0, pRM0). Pathological staging was pT3, pN1a, cM0, pStage IIIb. The patient recovered uneventfully and was discharged on POD 7 without complications.

## DISCUSSION

Laparoscopic colectomy in SIT has been increasingly reported; however, it remains technically demanding due to mirror-image anatomy. Reported challenges include non-dominant hand dissection in the deep pelvis for right-handed surgeons, limited instrument articulation, frequent collisions, and a high cognitive load to adapt to reversed orientation.^2–6)^ These factors often prolong operative time and increase technical complexity.

Robotic-assisted colectomy, however, offers distinct advantages that address many of these challenges. EndoWrist articulation facilitates precise dissection in confined spaces, while the stable 4th arm provides surgeon-controlled retraction, reducing reliance on assistants. Surgeon-controlled 3D visualization ensures stable exposure and eliminates delays associated with camera adjustment. Importantly, intuitive motion translation eliminates the need for mental reversal of anatomy, a major obstacle in laparoscopic SIT surgery.^7–9)^

In our case, preoperative 3D imaging enabled tailored port placement relative to the right ASIS, which, in combination with robotic articulation, minimized external and internal collisions, optimized instrument angles, and allowed intuitive, ergonomic console operation without spatial disorientation or intraoperative difficulties. These findings suggest that, when individualized port mapping is coupled with robotic articulation, the mirror-image anatomy of SIT does not necessarily translate into increased technical difficulty for experienced robotic surgeons. This underscores the importance of individualized port mapping in robotic colectomy for SIT.

A comparative summary of laparoscopic versus robotic-assisted colectomy in SIT is provided in **Table 2**, highlighting how robotic technology may overcome intrinsic laparoscopic limitations.

**Table 2:** Comparison of laparoscopic and robotic colectomy in SIT

Aspect	Laparoscopic approach (reported in SIT)^2–6)^	Robotic-assisted approach (current case and literature)^7–9)^
Surgeon orientation	Requires mental reversal of anatomy; increased cognitive load	Intuitive motion translation, eliminating need for mental reversal
Instrument handling	Limited articulation; non-dominant hand dissection often required	EndoWrist articulation enables precise dissection
Ergonomics	Awkward posture, surgeon fatigue	Stable ergonomics with seated console position
Exposure/retraction	Assistant-dependent; less stable	4 arm allows surgeon-controlled stable retraction
Instrument collisions	High risk due to mirror-image setup	Reduced by tailored port mapping and flexible docking
Visualization	2D or limited 3D; assistant-dependent camera	3D HD, surgeon-controlled camera with stable view
Reported outcomes	Prolonged operative times, higher technical difficulty	Feasibility demonstrated; improved dexterity and exposure

## CONCLUSIONS

Robotic-assisted colectomy can effectively overcome the ergonomic and technical challenges posed by SIT. Patient-specific port mapping, guided by preoperative imaging, is essential to maximize the benefits of robotic technology. This case adds to the limited evidence supporting robotic colectomy as a feasible and advantageous approach in SIT.

## DECLARATIONS

### Use of Artificial Intelligence (AI) tools

Some authors used ChatGPT (OpenAI, San Francisco, CA, USA), a generative AI tool, to assist with language refinement during manuscript preparation. The tool was not used to generate scientific content, analyze or interpret data, or modify images. All content was critically reviewed and verified by the authors, who take full responsibility for the accuracy and integrity of the manuscript.

## References

[1] Mayo CW, Rice RG. Situs inversus totalis: a statistical review of data on 76 cases with special reference to disease of the biliary tract. Arch Surg 1949; 58: 724–30. 18152884

[2] Miyasaka M, Teramura K, Kitashiro S, et al. Two cases of single-incision laparoscopic surgery for sigmoid colon and rectal cancer in situs inversus totalis. Surg Case Rep 2025; 11: cr.24–0016. 10.70352/scrj.cr.24-0016 PMC11946454 40151336

[3] Song SA, Liu S, Li F, et al. Rectal cancer with situs inversus totalis and previous malignant middle cerebral artery infarction: a case report and review of the literature. J Med Case Rep 2024; 18: 565. 39578876 10.1186/s13256-024-04903-7 PMC11585198

[4] Onishi R, Nomura A, Okada T, et al. Laparoscopic low anterior resection for rectal cancer with situs inversus totalis: surgical simulation using a video of laparoscopic right hemicolectomy - a video vignette. Colorectal Dis 2024; 26: 1315–6. 38561644 10.1111/codi.16975

[5] Zhang CY, He J, Huang LC, et al. Minimally invasive surgical treatment for sigmoid colon cancer in a patient with situs inversus totalis: a case report. Front Oncol 2025; 15: 1613470. 40909955 10.3389/fonc.2025.1613470 PMC12404946

[6] Yokoi R, Tajima JY, Kiyama S, et al. Usefulness of an intraoperative flipped monitor in laparoscopic surgery with situs inversus totalis: a case report of laparoscopic-assisted ileocecal resection. Surg Case Rep 2024; 10: 6. 38190089 10.1186/s40792-023-01806-5 PMC10774461

[7] Hara M, Ikeno Y, Kobayashi J, et al. Robot-assisted gastrectomy and sigmoid colectomy with situs inversus totalis: a case report. Surg Case Rep 2025; 11: cr.25–0073. 10.70352/scrj.cr.25-0073 PMC12230313 40625543

[8] Kato J, Hirokawa T, Kobayashi K, et al. Robotic hemi-colectomy for ascending colon cancer in a patient with situs inversus totalis. Surg Case Rep 2024; 10: 181. 39085519 10.1186/s40792-024-01982-y PMC11291778

[9] Kasai S, Hino H, Shiomi A, et al. Robotic-assisted surgery for rectal cancer with situs inversus totalis: a case report. Asian J Endosc Surg 2021; 14: 803–6. 33797194 10.1111/ases.12937

